# Therapeutic Effects of Natural Products on Cervical Cancer: Based on Inflammatory Pathways

**DOI:** 10.3389/fphar.2022.899208

**Published:** 2022-05-13

**Authors:** Zi-Wei Zhou, Hui-Zhi Long, Shuo-Guo Xu, Feng-Jiao Li, Yan Cheng, Hong-Yu Luo, Li-Chen Gao

**Affiliations:** ^1^ School of Pharmacy, University of South China, Phase I Clinical Trial Centre, The Affiliated Changsha Central Hospital, Hengyang Medical School, University of South China, Changsha, China; ^2^ Hunan Provincial Key Laboratory of Tumor Microenvironment Responsive Drug Research, Changsha, China

**Keywords:** inflammatory, cervical cancer, natural products, HPV, therapeutic

## Abstract

Inflammation is a protective response of the body to an irritant. When an inflammatory response occurs, immune cells are recruited to the injury, eliminating the irritation. The excessive inflammatory response can cause harm to the organism. Inflammation has been found to contribute to cervical cancer if there is a problem with the regulation of inflammatory response. Cervical cancer is one of the most common malignant tumors globally, and the incidence tends to be younger. The harm of cervical cancer cannot be ignored. The standard treatments for cervical cancer include surgery, radiotherapy and chemotherapy. However, the prognosis for this treatment is poor, so it is urgent to find a safer and more effective treatment. Natural products are considered excellent candidates for the treatment of cervical cancer. In this review, we first describe the mechanisms by which inflammation induces cervical cancer. Subsequently, we highlight natural products that can treat cervical cancer through inflammatory pathways. We also introduce natural products for the treatment of cervical cancer in clinical trials. Finally, methods to improve the anticancer properties of natural products were added, and the development status of natural products was discussed.

## 1 Introduction

Inflammation is the organism’s protective response to a pathogen or irritant. When an inflammatory response occurs, chemokines and cytokines are released, which activate innate immunity. Various immune cells are summoned to the site of injury. When the danger signal is removed, inflammation is programmed to be disabled. Inflammation could hurt the body if the original stimulus persists or if it cannot be controlled autonomously (Medzhitov, 2008; Fernandes et al., 2015). In short, inflammation is a double-edged sword. The organism needs to be able to regulate inflammatory responses according to actual conditions flexibly. Inflammation is one of the principal determinants of cancer, and the inflammatory response is also a dominant feature of cancer (Hanahan and Weinberg, 2011). Inflammatory cells are the prominent members of the tumor microenvironment, including macrophages, dendritic cells, neutrophils. Cancer cells could also release large amounts of cytokines and chemokines, which call in immune cells and aggravate inflammation again (Hemmat and Bannazadeh Baghi, 2019). And this demonstrates the vital link between inflammation and cancer. It has been found that chronic inflammation could increase the risk of cancer. For example, chronic bronchitis can increase the risk of lung cancer; the occurrence of pancreatic cancer can be induced by chronic pancreatitis; the number of helicobacter pylori is a principal determining factor of gastric cancer (Schetter et al., 2009; Sadri Nahand et al., 2020). Inflammation also plays a crucial role in the mechanism leading to cervical cancer.

Cervical cancer is the third leading cause of cancer-related death in women worldwide. The onset of cervical cancer is starting to get younger. It is a matter that cannot be ignored (Freddie et al., 2018; Olusola et al., 2019). As the leading cause of cervical cancer, HPV infects the epithelial cells of the cervix through sexual contact. The term “persistent human papillomavirus (HPV) infection” can be traced to two causes: an imbalance of the cervical-vaginal microbiome and an inflammatory response. It is the best condition for HPV infection (Zhou et al., 2021). In addition, as research continues, other microorganisms may also contribute to cervical cancer, such as *fusobacterium* spp., *mycoplasma genitalium*, *chlamydia trachomatis* and herpes simplex virus (HSV). They induce local inflammatory processes, but they also increase the chances of persistent HPV infection (Golais and Mrazova, 2020; Sudomova et al., 2021; Zhou et al., 2021). Although the immune system could clear most HPV infections, it will lead to an unfortunate beginning once HPV has completed its life cycle in the host cell (Kemp et al., 2010; Shafabakhsh et al., 2019). Immune cell infiltration can be caused by persistent HPV infection, contributing to cervical cancer if not diagnosed and treated correctly (Zheng et al., 2015). Patients with early-stage cervical cancer are commonly treated with surgical resection, while patients with advanced cervical cancer are treated with cisplatin-based chemotherapy and brachytherapy, both of which are usually administered simultaneously (Small et al., 2017). Neoadjuvant therapy needs further research. However, chemotherapy resistance and severe toxicity are also worrisome due to the poor prognosis (Kamran et al., 2022). Effective novel treatments need to be discovered in clinical settings. Plant-derived natural products might be used as candidates for new cancer medicines.

Natural products are considered a promising substitute for chemotherapy drugs or be used in combination with chemotherapy agents. Their wide range of sources, typical side effects, and diverse biological activities make them a popular target for researchers. There are ongoing studies demonstrating the non-negligible role of natural products in inhibiting cancer occurrence, development and spread (Mann, 2002; Ouyang et al., 2014; Thazin et al., 2017; Kikuchi et al., 2019). Therefore, this review first summarizes the relationship between inflammation and cancer, and a comprehensive description of cancer initiation, development and dissemination is provided. Next, we highlight the natural products for the treatment of cervical cancer based on inflammatory pathways, which can be divided into five categories: alkaloids, flavonoids, terpenoids, phenolic compounds, and others. We also focus on some natural products that have been treated before clinical treatment. We also discuss current approaches to improving the anticancer properties of natural products. Finally, we discuss the challenges and future directions of natural product development.

## 2 Liturature Search Strategy

We conducted a keyword search on articles published in the PubMed and Google Scholar database. The mechanism between cervical cancer and inflammation utilizes keywords such as “cervical cancer”, “inflammation”, “HPV” and “microbiome”. The drug summary section makes use of “flavonoids”, “alkaloids”, “phenols”, “terpenoids”, “natural products”, “paclitaxel”, “curcumin” or other related keywords. Most of the data cited in the 2010–2021 time frame for the most recent published study.

## 3 Inflammation is Linked to Cervical Cancer

### 3.1 The Role of Inflammation in the Initiation of Cervical Cancer

Tumorigenesis is an outcome controlled by many factors. Inflammation helps the organism fight off pathogens, but persistent inflammation could bring adverse effects, such as tissue damage. Repeated tissue repair and tissue damage can easily lead to DNA damage. The unstable mechanism is apt to be a promoter of tumorigenesis (Yamanishi et al., 2002). Neoplasms are characterized by epigenetic changes or mutations in oncogenic/tumor suppressor genes, as well as the transformation of normal cells. These two features are easily facilitated by persistent inflammation (Anuja et al., 2017). The complexity of the tumor approaches that of normal tissue. Tumor microenvironment (TME) refers to the environment surrounding tumor cells (Liu et al., 2020). TME consists of various cell populations, such as stromal cells, cancer cells, cancer stem cells (CSC), fat cells etc. In addition to cellular components, the extracellular matrix (ECM) contains a variety of signaling molecules. Immune inflammatory cells (ICs) also play an essential role in TME. One noteworthy phenomenon in TME is that immune cells are recruited due to the heterogeneity of the tumor microenvironment. At the same time, various pro-tumor and anti-tumor inflammatory cells fight each other (Hanahan and Weinberg, 2011). These cells are capable of releasing pro-inflammatory cytokines, chemokines and growth factors. DNA damage pathways can be activated by accumulating these molecules over time. Interleukin-1 (IL-1), IL-6, and IL-8 are involved in inflammatory processes, among which IL-1 and IL-6 play an important role in tumor cell growth and metastasis (Zhang et al., 2007). IL-1β is a common pro-inflammatory cytokine that can turn on immunosuppressive mechanisms and promote cancer development. It is also a marker molecule for identifying early cancer (Voronov and Apte, 2017). Notably, nuclear factor-kappa B (NF-κB) can be activated by both immune and cancer cells. Il-1β also activates NF-κB pathways (Lu et al., 2016; Yasuda et al., 2019). NF-κB is a nuclear transcription factor, and NF-κB signaling pathway plays a crucial driving role in innate and acquired immunity (Taniguchi and Karin, 2018). NF-κB may lead to chromosome instability and changes in epigenetics. NF-κB can induce mutations associated with mutations and increase the likelihood of genetic mutations. A study has identified that NF-κB could be able to induce cytidyl deaminase (CDA), which leads to the conversion of cytosine to thymine (Shimizu et al., 2012). At the same time, NF-κB and other transcription factors such as signal transducer and activator of transcription (STAT3) express chemokines that induce more aggregation of inflammatory cells, further increasing the severity of inflammation (Karin and Lin, 2002; Sparmann and Bar-Sagi, 2004).

**FIGURE 1:**
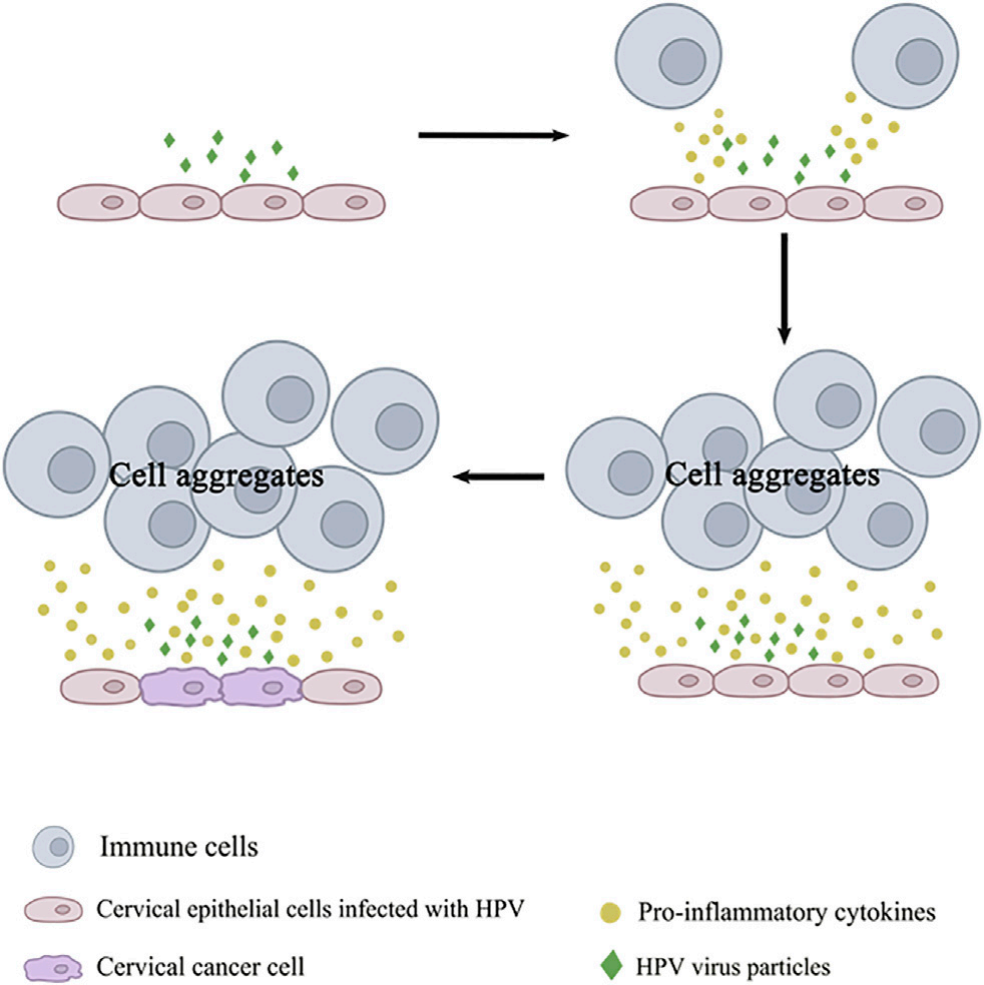
The process by which HPV infected cells becomes cancerous. The process by which HPV infected cells becomes cancerous. HPV virus particles are released when HPV infected cells undergo lysis. At this point, immune cells are recruited by virus particles and release pro-inflammatory cytokines. Inflammation will persists if the virus particles are not completely removed in time. This could cause HPV infected cells to become cancerous.

Among the contributing factors to cancer development, oxidative damage is a non-negligible factor in cancer promotion. Various inflammatory cells clustered in the inflammatory site can cause the accumulation of reactive oxygen species (ROS) and nitrogen oxides while releasing pro-inflammatory cytokines. Neutrophils are one of the main sources of ROS (Nicolás-ávila et al., 2017). Oxygen nitrification stress is positively related to chronic inflammation. ROS and nitrogen oxides have genotoxic effects, inducing DNA damage. Genetic aberrations could be caused by this change in DNA (To et al., 2020; Liskova et al., 2021b). The likelihood of important genetic mutations is increased by the presence of these factors, which are the occurrence of inflammatory cytokines and chemokines, the occurrence of oxygen nitrification and the repeated damage and repair of the tissue. P53 is a common and vital gene that plays an important role in associated tumors caused by chronic inflammation (Yamanishi et al., 2002). Persistent HPV infection is an essential factor of cervical cancer. When the balance of the reproductive tract microbiota is disturbed, local inflammatory responses are activated. The endometrial epithelial barrier of the cervix is disrupted due to changes in the products of the reproductive tract and the persistence of inflammation. It makes HPV infection much more accessible. The invasion of specific pathogens could also lead to this trend. For example, HSV-2 can be a cofactor of HPV. There is localized ulceration of the cervix, which in the case of HSV-2 infection facilitates the entry of HPV into the basal cells. As expected, HSV-2 induces inflammation, leading to cytotoxic effects that promote DNA mutations (Golais and Mrazova, 2020). During infection, HPV integrates its genetic information into the DNA of the host cell, causing a series of effects that are conducive to its survival and promote cancer in the host cell. HPV attaches to the plasma membrane of keratinocytes as it travels through the host (Adefuye and Sales, 2012). It cannot be detected by the innate immune system and is one of the mechanisms HPV evades detection. When infection occurs, cytotoxic mechanisms are activated. Some immune receptors such as major histocompatibility complex Ⅰ (MHC Ⅰ) and MHC Ⅱ intervene and are recognized by NK cells (Benyue et al., 2003; Ashrafi et al., 2006; Miura et al., 2010). HPV E5 oncoprotein interferes with the expression and transport of these key immune receptors, thus preventing the immune system from recognizing infected cells (Dimaio and Petti, 2013). It affects antigen presentation and contributes to the persistence of viral infection. Furthermore, the HPV18 E5 protein is also required for viral DNA synthesis in basal cells (Wasson et al., 2017). In addition, pattern-recognition receptors (such as Toll-like receptor 9) are downgraded by HPV, turning off the interferon pathway. At this point, the chance of integrating the host cell DNA of HPV is significantly increased. When the viral oncogenes are successfully integrated into the host cell DNA, the host cell automatically synthesizes the viral DNA and releases more viral particles as the cell matures, migrates and apoptosis (Adefuye and Sales, 2012). Innate and acquired immunity is activated. However, there are also cases where inflammation is exacerbated by untimely pathogen clearance and contributes to malignant lesions (Tindle, 2002). The carcinogenesis of infected cells is shown in Figure 1. After keratinocytes are successfully infected with HPV, the viral E1 and E2 proteins begin to play, which leads to the formation of viral DNA loops (Kadaja et al., 2009). At the same time, oncogenes E6 and E7 come into play, prompting the release of pro-inflammatory cytokines and inducing persistent inflammation (Liu et al., 2015). After persistent infection caused by HPV, immune cells are activated and aggregated, and the accumulation of cytokines and ROS causes tissue damage. High expression of IL-10 and transforming growth factor β1 (TGF-β1) also causes the entire tumor microenvironment to receive immunosuppression (Wang et al., 2018).

**FIGURE 2:**
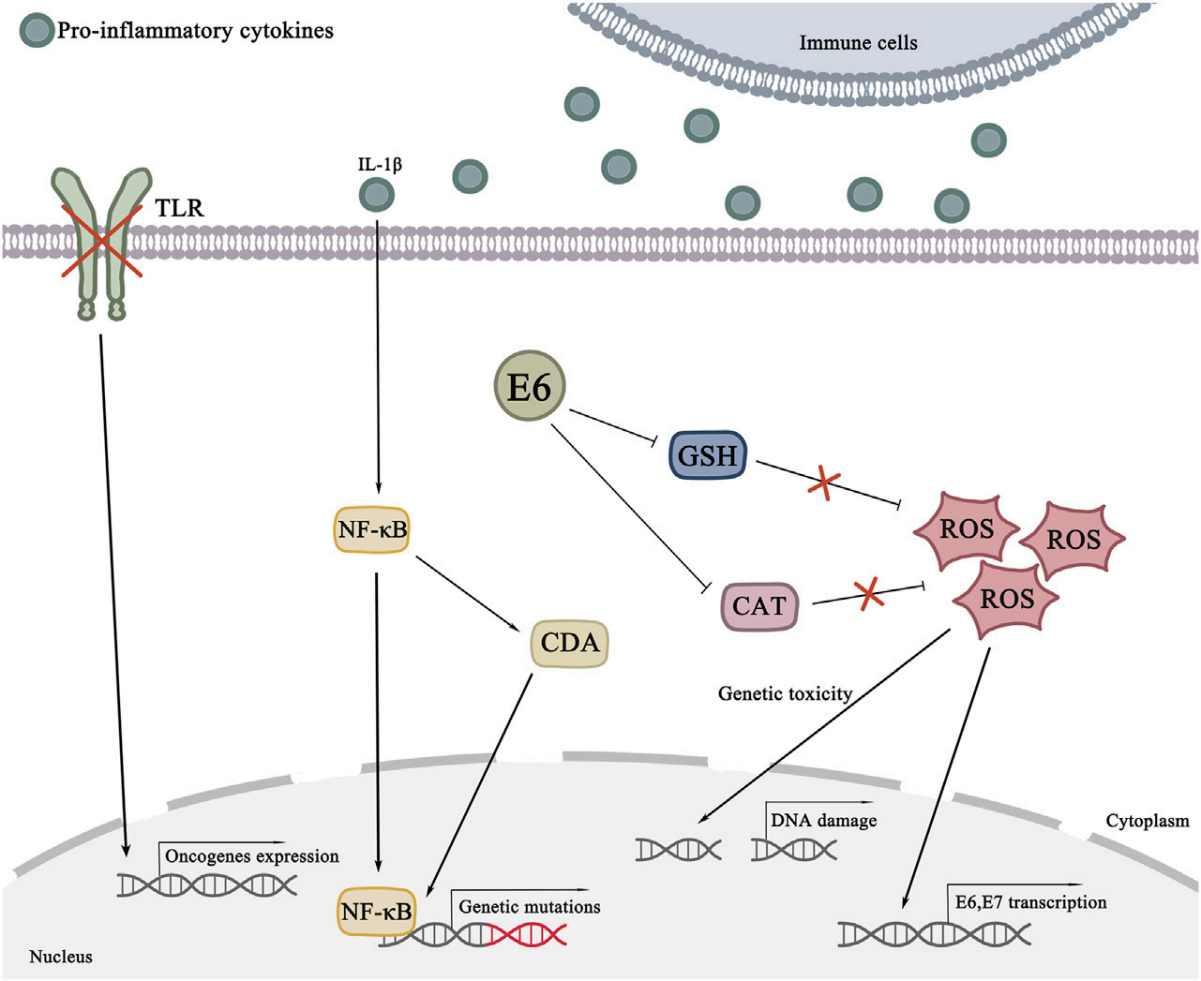
Signaling pathways in the initial stage of cervical cancer cells. NF-κB is activated by pro-inflammatory cytokines such as IL-1β. NF-κB can not only change cytosine into thymine, but also induce CDA expression, thereby inducing gene mutation. The accumulation of inflammatory cells allows ROS to accumulate. The buildup of ROS could cause DNA damage. HPV E6 oncoprotein can inhibit GSH and CAT. ROS is further accumulated.

Interestingly, E2 oncoprotein is lost when the HPV gene is integrated, which can be used as a marker for cervical cancer. E6 and E7 oncoprotein are activated when E2 oncoprotein is lost. In the study by Prabhavathy et al. E2 inhibited the expression of the E6 gene, suggesting that E2 oncoprotein plays a significant role in tumor suppression (Prabhavathy et al., 2015). Oxidative damage can be caused by persistent infection with HPV. The damaged antioxidant mechanism is unable to strike a balance with a large amount of oxynitrogen compounds. The chances of cancer cells appearing in this imbalance are greatly increased. Oncogene E6 activates glutathione (GSH) and catalase (CAT), so the appearance of damage and mutation of normal cellular DNA becomes reasonable (Hemmat and Bannazadeh Baghi, 2019; Preci et al., 2021). Notably, nitric oxide (NO) also induces transcription of oncogenes E6 and E7, suggesting a mutually reinforcing mechanism between HPV infection, inflammatory response and oxygen nitration response (Bogdan, 2001). The signaling pathways of the initial stage of cervical cancer cells can be referred to in Figure 2.

### 3.2 The Role of Inflammation in the Development of Cervical Cancer

In addition to immune cells, tumor cells are capable of producing their own pro-inflammatory cytokines and growth factors after formation, which further drive large numbers of immune cells together, such as neutrophils and monocytes. Monocytes in the tumor inflammatory microenvironment are able to differentiate into tumor-associated macrophages (TAMs) (Adefuye and Sales, 2012). TAMs can produce proteases such as cysteine cathepsin and activate cytokines, undoubtedly contributing to tumor development (Quail and Joyce, 2013). Macrophages can be divided into M1 and M2. In early-stage tumors, M1 macrophages predominate and recruit natural killer cells, whereas in advanced tumors, macrophages transform into M2 phenotype capable of remodeling tissue and angiogenesis. As a result, TAMs are beneficial to the proliferation and survival of tumor cells (Biswas and Mantovani, 2010; Liu et al., 2020).

Hypoxia is a common phenomenon in the inflammatory microenvironment of tumors due to the transformation of monocytes into macrophages, massive infiltration of immune cells, and vascular structure disorder. Hypoxia-inducible factor-1α (HIF-1α) is activated, and the survival of tumor cells is guaranteed to some extent. Cytokines like tumor necrosis factor-α (TNF-α) and IL-1β can also promote the proliferation of cancer cells (Gilkes and Semenza, 2013; Zhang et al., 2021). Moreover, NF-κB not only blocks tumor cell apoptosis induced by oncogene Ras and regulates the transcription of anti-apoptotic genes (Zhang et al., 2003).

In the process of cervical lesions, the role of various cytokines still should not be underestimated. In the presence of ROS, the HPV16 E5 protein can promote the degradation of the proteasome of Bax, thereby inhibiting the apoptosis of cervical cancer cells (Oh et al., 2010). E5 also inhibits Fas ligand (FasL) and tumor necrosis factor-associated regulation to ligand (TRAIL) (Kabsch and Alonso, 2003). In addition, The high levels of E6 and E7 oncoproteins overexpress IL-16, which activates the NF-κB pathway and promotes the proliferation of cancer cells (Qiongying et al., 2018). By binding to p53, E6 oncoprotein promote DNA mutations in normal cells, thus inhibiting apoptosis of cancer cells (Hemmat and Bannazadeh Baghi, 2019). Dysregulation of JAK/STAT signaling has also been shown to contribute to cancer progression. The STAT pathway is activated when cytokines (such as IL-6) and growth factors bind to transmembrane receptors. This leads to JAK actication, followed by recruitment, phosphorylation, and activation of STAT proteins. Subsequently, the STAT dimer is transferred to the nucleus and binds to target genes (Scarth et al., 2021). In fact, E5 oncoprotein also induces phosphorylation of STAT in cervical cancer cells and may be regulated by activated EGFR (Akerman et al., 2001; Spangle et al., 2013).

**FIGURE 3:**
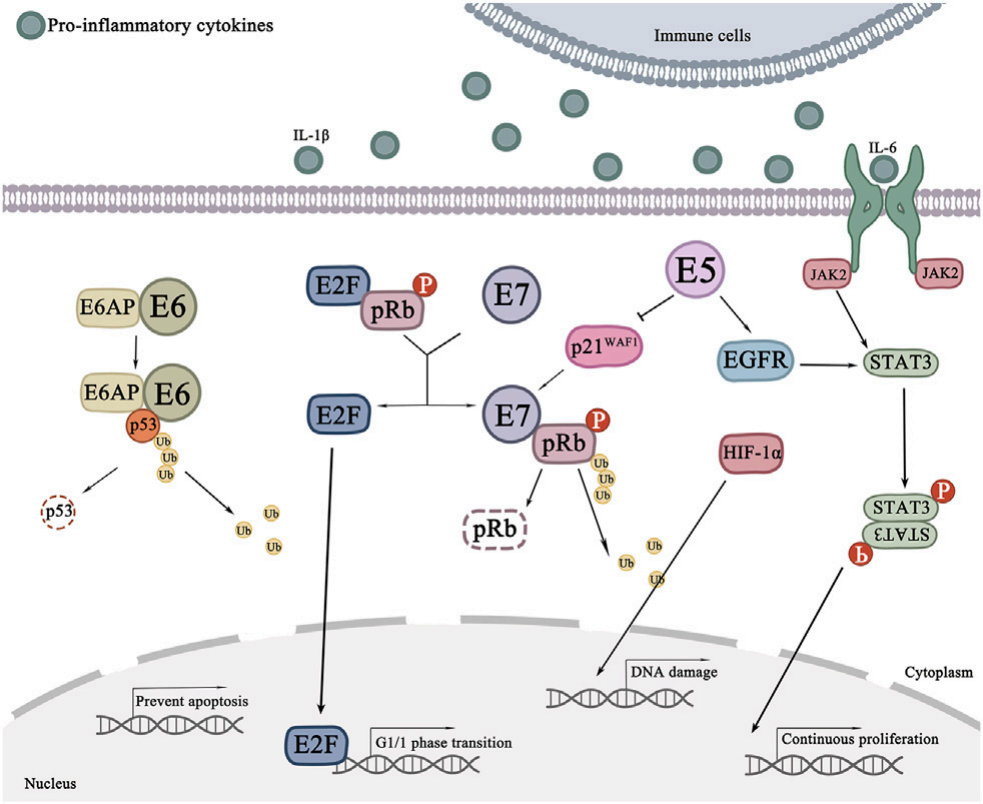
Signaling pathways in the development stage of cervical cancer cells. E7 oncoproteins binds to the PRB-E2F complex, a step that separates E2F from pRb. In the case of high E2F expression, cells will pass the G1/S phase and pRb will eventually be degraded by the proteasome. The STAT3 signaling pathway can be activated by IL-6 to promote cell proliferation which can also be induced by HIF-1α, E5 can inhibit the expression of p21^WAF1^, thus promoting the separation of E2F from pRb, and regulate STAT3 by regulating EGFR.

Processes such as cell cycle arrest, apoptosis and DNA damage responses can be regulated by p53. HPV induces p53 ubiquitination by forming a complex between p53, E6 oncoprotein, and E6-associated protein (E6AP). P53 is degraded, contributing to chromosome instability. This is one of the mechanisms of cervical cancer cells to avoid their apoptosis and cycle arrest (Medda et al., 2021). E6, one of the culprits in transforming normal cells into tumor cells, degrades and inhibits p53 via promoting ubiquitination. E7 binds to retinoblastoma protein (pRb) (Parida and Mandal, 2014). PRb is a tumor suppressor protein that works with P107 and P130 to form a “pocket protein” that regulates the cell cycle. Additionally, pRb can also bind to E2F transcription factors to form a complex, promoting cell arrest in the G1/S phase, thus regulating the rhythm of cell growth and division cycle. In cervical cancer cells, E7 oncoproteins bind to the pRb-E2F complex, a step that separates E2F from pRb. In the case of high E2F expression, cells will pass the G1/S phase and pRb will eventually be degraded by the proteasome (Scarth et al., 2020). On the other hand, p21^WAF1^ protein controls phosphorylation of PRB-E2F complex and transcription of genes that regulate cell proliferation (Lim and Kaldis, 2013). E5 protein can inhibit p21^WAF1^ gene expression (Tsao et al., 1996), which activates the cyclin D/CDK4 complex, which in turn promotes the release of transcription factors that release E2F, leading to cell cycle changes. Collectively, pRb is the guard that ensures average cell growth and differentiation (Hanahan and Weinberg, 2011). Due to the inactivation of tumor suppressor genes P53 and pRb, cells caused by HPV have a much higher chance of becoming cancerous. Furthermore, E6 and E7 oncoproteins inhibit CDKs inhibitors and disrupt the control of cell cycle checkpoints. The signaling pathways of the development stage of cervical cancer cells can be referred to in Figure 3.

### 3.3 The Role of Inflammation in the Metastis of Cervical Cancer

Tumor metastasis involves two aspects: the invasion and invasion of cancer cells to surrounding tissues, and the other is an epithelial-mesenchymal transition (EMT) (Varga and Greten, 2017; Greten and Grivennikov, 2019). As the main force of tumor metastasis, cancer stem cells (CSCs) play a crucial role. CSCs bind to various cytokines and chemokines, such as IL-6, IL-8, TGF-β, and vascular growth factors. These molecules regulate the metastasis of tumor cells. The complex regulatory network between different cells can be interfered with by CSCs. At the same time, CSCs had a closer transcription behavior with mesenchymal cells than with normal epithelial cells (Babashah, 2015; Hong et al., 2018; Powell et al., 2020). Further, inflammatory cells likewise play a role in tumor invasion and spread. Endoosmosis and extravasation are characteristic features of tumor diffusion in blood vessels and lymphatics, which are regulated by specific adhesion molecules and integrins. Coincidentally, pro-inflammatory cytokines can induce the expression of adhesion molecules and integrins. Due to a series of abnormal biological effects of tumor cell regulation, adhesion between cells also becomes extremely smooth (Fu et al., 2020). NF-κB regulates some EMT molecules (such as Smad-interacting protein1) which initiate EMT and enhance cancer cell migration (Scheel and Weinberg, 2012; Pires et al., 2017). Furthermore, NF-κB and inflammatory cells directly regulate the expression of transfer-related genes (Malki et al., 2021). NF-κB could also induce self-renewal and metastasis in CSCs (Rinkenbaugh and Baldwin, 2016). TGF-β also plays a significant role in tumor metastasis, where it can be produced by cancer cells and affects the differentiation of Treg and TH17 cells. Some studies have found that TGF-β has an effect in tumor metastasis and invasion (Meulmeester and Dijke, 2011). Hypoxia is known to be a characteristic feature of the tumor microenvironment, and HIF-1α is activated in the presence of both hypoxia and inflammation, which leads to the activation of vascular endothelial growth factor (Kamura et al., 1999). Inflammatory media and proteases can also be triggered by HIF (Agnieszka and Maciej, 2015).

The development and spread of cervical cancer also follow these typical cancer development rules. IL-1 adhesion to vascular endothelial cells promotes extravasation and tumor invasion (Hemmat and Bannazadeh Baghi, 2019). IL-6 phosphorylates STAT3 and induces STAT3 to promote the invasion of cervical cancer cells by activating the transcription of matrix metalloproteinase (MMPs) (Xie et al., 2004). Besides, STAT3 directly binds to focal adhesion kinases (FAK) and paxillin, promoting cancer cell invasion. It has been shown that a high expression of FAK exists in HPV-infected patients. Interestingly, E6 oncoprotein binds to paxillin and fibulin1 (Du et al., 2002; Deivendran et al., 2014). COX-PG pathway is one of the key signaling pathways between inflammation and cancer. E5 oncoprotein regulates the COX-PG pathway (Sales et al., 2002; Libra, 2009; Adefuye et al., 2014). Some studies have found that COX-2 expression is positively correlated with MMPs (Liu et al., 2015). Meanwhile, activation of EGFR signaling pathway can increase COX-2 expression (Hemmat and Bannazadeh Baghi, 2019). Coincidentally, remodeling of the extracellular matrix (ECM) can be induced by MMP, which promotes angiogenesis. HPV E5 protein can up-regulate the expression of COX-2 and MMP-7, and it has been proved that E5 protein can promote the invasion and spread of cervical cancer by activating the NF-κB and EGFR pathways (Kemp et al., 2010; Gutierrez-Xicotencatl et al., 2021).

## 4 The Rapeutic Potential of Natural Products Through Inflammation Pathways

Human beings have never stopped the exploration of cancer. With the continuous in-depth exploration of cancer, more possibilities have been brought to treatment. Finding better drugs with fewer side effects has been the goal of many researchers over the years. Concurrently, with the gradual deepening of biochemistry and pharmacology, scholars have gradually paid attention to natural products. More and more research has begun to be carried out in the direction of natural products in the treatment of cervical cancer. Nature, a treasure trove of medicines, is a source of many natural medicines that can be used to treat and prevent disease, especially in the field of cancer (Dias et al., 2012). In addition, natural products are also considered to be evolutionarily optimized ligands for biological targets and receptors (Grigalunas et al., 2020). We described 30 natural products that have the potential to treat cervical cancer by influencing a range of inflammatory effects.

### 4.1 Alkaloids

A study explores the ability of *piperine* (PP) to reverse the resistance of cervical cancer cells. PP is a pepper and alkali compound extracted from *Piper longum* L. It inhibits oxidative stress, inflammatory response, and even tumor growth. The expression of p65 was significantly reduced after the low concentration of PP treated Hela cells. PP decreased the expression of p-STAT3, NF-κB, and Bcl-2 in HeLa cells, while the activities of Bax, Bid, Caspase and PARP were increased (Han et al., 2017). In addition, in Dasari’s study, they used an *in vitro* model to study the effects of *neptoline* on HeLa and SiHa cells. The results show that *neptoline* inhibited the activity of HeLa and SiHa cells and increased the intracellular ROS, which in turn promoted autophagy and apoptosis of cervical cancer cells in a dose-response manner. Meanwhile, *Nephrine*, as a lotus seed alkaloid, is also very toxic to normal cells (Dasari et al., 2020). In addition to influencing the growth of cervical cancer cells by affecting signaling pathways and oxidative stress, some alkaloids can directly affect gene transcription and protein synthesis of HPV. After *berberine* is absorbed by HeLa cells, the tubulin network of HeLa cells is destroyed. *Berberine* is destroyed by the microtubule protein network of Hela cells absorbed by HeLa cells. In addition, the E6/E7 expression of HeLa cells that absorbed *berberine* is also significantly suppressed. The mechanism is that *berberine* can further adjust the expression of the cancer gene p53 and further regulate the HPV 18 E6/E7 virus carcinoma. Via western blot analysis, the expression of Cyclin and NF-κB were also decreased, suggesting that *berberine* may also be involved in the treatment of cervical cancer through the signal transduction pathway (Saha and Khuda-Bukhsh, 2014). Another study also found that *berberine* can selectively inhibit AP-1 group activation, thereby down-regulating HPV oncogene expression (Mahata et al., 2011). *Colchicine*, a plant-derived alkaloid, could significantly reduce the expression of HPV 16 E6/E7 mRNA and protein in CaSki and HeLa cells. This effect results in up-regulation of tumor suppressor proteins p53 and Rb and down-regulation of phosphorylated Rb (pRb) proteins (Yan et al., 2020).

### 4.2 Flavoniods

Flavonoids are widely found in plants in nature. Studies have found that flavonoids have antimicrobial, antioxidant, anti-inflammatory and anti-tumor effects. Flavonoids are also an essential dietary component of human beings (Serafini et al., 2010; Panche et al., 2016; Choy et al., 2019; Maleki et al., 2019). *Icaritin* can increase the ROS expression in the Hela cells, increasing the number of DNA fractures in the HeLa cells, raising the expression of Bax and Caspase 3 and 9 (Xin et al., 2018). In another study, *morusin* decreased the expression of NF-κB, p65, and Bcl-2 and increased the levels of Bax and Caspase-3 (Wang et al., 2013). *Wogonin* had cytotoxic effects on both SiHa and CaSki cells. The oncogenes of E6 and E7 virus were significantly inhibited in *wogonin*-treated SiHa and CaSki cells. Besides, *wogonin* can cleave poly ADP ribose polymerase (Kim et al., 2013). *Kaempferol-7-O-b-D-glucoside (KG)* can reduce the nuclear translocation of NF-κB in a dose-response manner. At the same time, *KG* can also up-regulate the expression of Bax and down-regulate Bcl-2 (Xu et al., 2008). Similarly, *fisetin* could inhibit the p38MAPK-dependent NF-κB signaling pathway in a concentration-dependent manner. *fisetin* can also down-regulating the expression of urokinase-type plasminogen activators (Chou et al., 2013). *Baicalein*, extracted from the root of *Scutellaria baicalensis* Georgi, promotes HeLa cell apoptosis by inhibiting the phosphorylation of NF-κB and I-κBα, thereby blocking the TNF-α induced nuclear ectopia of P65. In addition, *baicalein* was found to reduce the expression of pro-inflammatory cytokines such as IL-8 and monocyte chemoattractant protein 1 (MCP1) (Xiaolan et al., 2014; Yong et al., 2015). *Fisetin* is widely found in various vegetables and fruits, and nuts are no exception (Kashyap et al., 2019). The evidence from a study suggests that *fisetin* inhibits the invasion of SiHa and CaSki cells by inhibiting the phosphorylation of P38/MAPK, affecting NF-κB and inhibiting nuclear translocation (Chou et al., 2013). *Luteolin* has been found to have a variety of therapeutic effects. *Luteolin* could inhibit the activation of NF-κB by inhibiting TNF-α, and enhance the activity of JNK, thus promoting the apoptosis of HeLa cells (Shi et al., 2004). Similarly, as reported, *naringin* also promoted the apoptosis of HeLa cells by decreasing the expression of NF-κB and COX-2 (Zeng et al., 2014). *Puerarin* can be extracted from *Pueraria alopecuroides* Craib, and its anti-inflammatory effects have been demonstrated in various disease models. *Puerarin* increases the activity of IL-2 and superoxide dismutase (SOD) in plasma of U14 cervical cancer mice. Excess free radicals were removed in certain doses. *Puerarin* as reported improved the tissue damage induced via ROS, then increased the ability to fight tumors (He et al., 2021).

### 4.3 Terpenoids

Terpenoids are the most extensive group of plant components. These compounds have been reported to possess a variety of pharmacological activities, such as anticancer activity (Cox-Georgian et al., 2019; Kamran et al., 2022). *Triphala* inhibits the phosphorylation of NF-κB, decreases the expression of cyclin D1, and increases the expression of p53 at doses. The result is that the proliferation of HeLa cells is inhibited (Zhao et al., 2018). TNF related apoptosis inducing ligand (TRAIL) plays an essential role in apoptosis. On the one hand, *artesunate* inhibits NF-κB activation, thereby reducing the expression of pro-survival proteins such as XIAP. This can enhance the pro-apoptotic effect of TRAIL and promote the apoptosis of HeLa cells (Thanaketpaisarn et al., 2011). On the other hand, *artesunate* also inhibit the expression of COX-2, which inhibits the proliferation of HeLa and CaSki cells. The percentage of T cells is also decreased due to the absorption of *artesunate* (Zhang LX. et al., 2014).

### 4.4 Phenols

The research of Manickam et al. has shown that *Curcumin* has the ability to regulate apoptosis, proliferation and angiogenesis. They suggest that curcumin does this by regulating the expression of kinases and gene factors that inhibit the expression of NF-κB. This speculation was finally confirmed in Hela cells (Manickam et al., 2005). In addition, *Curcumin* has attracted much attention because of its extensive anti-cancer, anti-oxidation, anti-inflammatory, anti-bacterial and other therapeutic effects (Vecchione et al., 2016). *curcumin* has excellent anti-inflammatory activity because it could inhibit the proliferation of HeLa, CaSki and Siha cells by inhibiting the expression of COX-2 and inducible nitric oxide synthase (INOS) (Singh and Singh, 2009). *Camellia sinensis* (L.) Kuntze is a popular herb with many biologically active natural products, such as (-) *Epigallocatechin gallate (EGCG)*, (-) *EpigalLocatechin 3-gallate (ECG)*, (-) *Epigallocatechin (EGC)*, and (+)*catechin*. Among them, *EGCG* is well known for its various pharmacological activities. Being a catechin compound, on the one hand, EGCG inhibits the activation of NF-κB in HeLa cells and SiHa cells, thereby inhibiting the expression of COX-2 (Singh et al., 2011; Mp et al., 2021). On the other hand, EGCG can regulate the number of ROS, indicating that EGCG can play an anti-tumor role through the antioxidant pathway (Min and Kwon, 2014). *Quercetin* is a dietary compound that is often found in vegetables and fruits as a secondary plant metabolite. It has a variety of biological activities (Andres et al., 2018). A finding of a study suggests that *quercetin* can target the NF-κB pathway and inhibit HeLa cells proliferation. Furthermore, *quercetin* also inhibits the binding of the oncoprotein E6 to E6AP, which removes the fear of p53 being unused, thereby, inducing apoptosis of HeLa and SiHa cells (Vidya Priyadarsini et al., 2010; Clemente-Soto et al., 2019). *Resveratrol* is a dietary polyphenol derived from grapes, berries and other plants. *Resveratrol* has been shown to inhibit the migration of HeLa cells by inhibiting NF-κB and MMP9 expression (Tang et al., 2007; Zhao et al., 2019). Further, Zhang et al.'s research team treated different cervical cancer cell lines with *resveratrol* to explore the effects of Resveratrol on STAT3, Notch and Wnt pathways. It was concluded that *resveratrol* could simultaneously inhibit the action of three signaling pathways, thereby promoting the apoptosis of HeLa and SiHa cells (Zhang P. et al., 2014). *Kaempferol* is found in various fruits and vegetables, such as onions, parsley and oranges. *In vitro* studies, it can inhibit nuclear heterotopic of NF-κB in C33A, CaSki, HeLa and SiHa cells, and then promote cell cycle arrest in G2/M phase (Marius et al., 2016; Souza et al., 2017; Wozniak et al., 2021). *Morin* is widely derived from a variety of fruits and vegetables (Osage orange, apple guava, strawberry, almond shell, sweet chestnut, onion, and jackfruit). Morin decreases NF-κB mRNA expression and promotes HeLa cell apoptosis. In addition, *morin* could increase ROS expression in cancer cells (Zhang et al., 2018; Solairaja et al., 2020). *Rutin* is an active substance in asparagus, buckwheat, apricot, apple, cherry, grape and other plants. Several lines of evidence suggest that *rutin* can reduce the expression of COX-2 and the leukocytes invasion when rutin is injected into K14-HPV16 mice according to the prescribed dosage (Deepika et al., 2018; Moutinho et al., 2018; Nouri et al., 2020). Derived from the dry root of scutellaria baicalensis. *Salvianolic Acid B* can reduce the expression level of tumor necrosis factor TGF in Hela cells, thus promoting cell apoptosis (Jing et al., 2016).

### 4.5 Others

It has been reported that E6-associated E3 ubiquitin ligase E6AP targets p53 degradation and E7-associated transcription factor E2F1 is also decreased by *tanshinone IIA* in a dose-dependent manner (Munagala et al., 2015). After *emodin* treatment of SiHa and C33A cells, intracellular HOCl/OCl^−^ was decreased, p-Akt activation was also inhibited as well as NO^−^, O_2_^−^ in a dose-response manner (Moreira et al., 2018). *Eugenol* could regulate cell viability. After *eugenol* treatment, the expressions of PARP, Bax, Caspase-3 and ROS were up-regulated, while the expression of Bcl-2 was down-regulated in HeLa and SiHa cells (Das et al., 2018). The *ethyl acetate extracts isolated from pistacia vera L* could induce apoptosis and inhibit angiogenesis. Studies have found that it can down-regulate the expressions of TNF, Bcl-2, IAP and TRAF in a dose-response manner (Seifaddinipour et al., 2018). *Praeruptorin-B* has been reported to have excellent antitumor activity. *Praeruptorin-B* can down-regulate NF-κB, MMP-2 and -9 in HeLa and SiHa cells. Interestingly, *praeruptorin-B* could block Akt phosphorylation without affecting the MAPK pathway (Hung et al., 2019). Similarly, *Praeruptorin-B* would down-regulated the expression of NF-κB, MMP-2 and MMP-9, which affected the proliferation of HeLa and SiHa cells. In addition, *praeruptorin-B* inhibits Akt phosphorylation, thus inhibiting cell invasion (Hung et al., 2019).

Up to now, the anti-tumor mechanism of natural products mainly involves multiple molecular mechanisms, such as promoting cell apoptosis, regulating gene transcription and protein synthesis, and regulating cell signal transduction pathways, which can also contribute to the treatment of inflammation-related cancers, indicating the direction. As for microbiota regulation, most of the current literature mainly directly affects the dynamic balance of microbiota through probiotics. Consequently, we believe that natural products might be an emerging therapeutic direction. Whether natural products can directly affect the entire cervix vaginal microbiota remains to be investigated. We summarized the experimental cell, time/dose development and mechanism in natural product research in Table 1, providing a reference for readers.

**TABLE 1:** Therapeutic potential of natural products based on inflammation in cervical cancer.

Name	*In-vitro/vivo* odels	Mechanism	Dose/time-effect	Ref
Colchicine	CaSKi and HeLa cells	Decrease the expression of HPV 16 E6/E7 mRNA and protein, increase	Dose-effect	Yan et al. (2020)
Piperine	HeLa cells, mice xenograft models	Reduce the expression of p65, decreased the expression of p-STAT3, NF-κB, and Bcl-2 in HeLa cells, while the activities of Bax, Bid, Caspase and PARP were increased	Dose-effect	Han et al. (2017)
Neferine	HeLa and SiHa cells	Increase the intracellular reactive oxygen species (ROS)	Dose-effect	Dasari et al. (2020)
Berberine	HeLa cells	Decrease the express of HPV 18 E6/E7, cyclin and NF-κβ. inhibit AP-1 group activation	Dose-effect	(Mahata et al., 2011
				Saha and Khuda-Bukhsh, (2014)
Icaritin	HeLa cells	Increase the expression of ROS, the number of DNA fractures, raise the expression of Bax and Caspase 3 and 9	Dose-effect	Xin et al. (2018)
Morusin	Human cervical CSCs	Decrease the expression of NF-κB, p65, and Bcl-2, and increased the levels of Bax and Caspase-3	Dose-effect	Wang et al. (2013)
Wogonin	SiHa and CaSki cells	Decrease the express of HPV 18 E6/E7, cleave poly ADP ribose polymerase	Dose-effect	Kim et al. (2013)
Kaempferol-7-O-b-D-glucoside	HeLa cells	Reduce the nuclear translocation of NF-κB, upregulate the expression of Bax and down-regulate Bcl-2	Time-effect, dose-effect	Xu et al. (2008)
Fisetin	SiHa and CaSki cells	Inhibit the p38MAPK-dependent NF-κB signaling pathway	Dose-effect	Chou et al. (2013)
Tanshinone IIA	CaSki cells	Decrease in HPV16 E6 and E7 protein levels	Time-effect, dose-effect	Munagala et al. (2015)
Emodin	SiHa cells, C33A	Inhibit the NO-,O2- and p-Akt activation, decrease HOCl/OCL^-^	Dose-effect	Moreira et al. (2018)
Eugenol	HeLa and SiHa cells	Up-regulate the expression of PARP, Bax, Caspase-3 and ROS, down-regulate the expression of Bcl-2	Time-effect	Das et al. (2018)
Ethyl acetate extracts isolated from Pistacia vera L	CaSki cells	Down-regulate the expressions of TNF, Bcl-2, IAP and TRAF	Time-effect, dose-effect	Seifaddinipour et al. (2018)
Praeruptorin B	HeLa and SiHa cells	Down-regulate NF-κB, MMP-2 and -9	Dose-effect	Hung et al. (2019)
Baicalein	HeLa cells	Inhibit the IL-8, phosphorylation of NF-κB and I-κBα, blocking the TNF-α induced nuclear ectopia of p65	Dose-effect	(Xiaolan et al., 2014
				Yong et al. (2015)
Fisetin	SiHa and CaSki	Inhibit the phosphorylation of P38/MAPK, affecting NF-κB and inhibiting nuclear translocation	Dose-effect	Chou et al. (2013)
Luteolin	HeLa cells	Inhibit the activation of NF-κB by inhibiting TNF-α, and enhance the activity of JNK	Time-effect, dose-effect	Shi et al. (2004)
Naringin	HeLa cells	Decrease NF-κB and COX-2	Time-effect, dose-effect	Zeng et al. (2014)
Puerarin	U14 cervical cancer mice	Increase the activity of IL-2 and superoxide dismutase (SOD)	Time-effect	He et al. (2021)
Triphala	HeLa cells	Inhibit the phosphorylation of NF-κB, decrease the expression of cyclin D1, and increased the expression of p53	Dose-effect	Zhao et al. (2018)
Artesunate	HeLa cells	Inhibit NF-κB	Time-effect, dose-effect	Thanaketpaisarn et al. (2011)
	HeLa and CaSki cells	Inhibit the expression of COX-2	Dose-effect	Zhang et al. (2014a)
Curcumin	HeLa, CaSki and SiHa cells	Inhibit the expression of NF-κB, COX-2 and INOS	Dose-effect	Manickam et al. (2005)
EGCG	HeLa and Siha cells	Inhibit the activation of NF-κB, the expression of COX-2	Dose-effect	(Singh et al., 2011
				Mp et al. (2021)
		Regulate the number of ROS	Time-effect. dose-effect	Manohar et al. (2013)
Quercetin	SiHa and HeLa cells	Inhibits the binding of the oncoprotein E6 to E6AP	Dose-effect	(Vidhya Priyadarsini et al., 2010
				Clemente-Soto et al. (2019)
Resveratrol	HeLa cells	Inhibiting NF-κB and MMP9 expression	Dese-effect	Tang et al. (2007)
Kaempferol	C33A,CaSki, HeLa and SiHa cells	Inhibit nuclear heterotopic of NF-κB, promote cell cycle arrest in G2/M phase	Time-effect	Xu et al. (2008)
Morin	HeLa cells	Decrease NF-κB mRNA expression, increases ROS expression	Dose-effect	(Zhang et al., 2018
				Solairaja et al. (2020)
Rutin	K14-HPV16 mice	Reduce the expression of COX-2 and the leukocytes invasion	Time-effect, dose-effect	(Deepika et al., 2018
				Moutinho et al., 2018
				Nouri et al. (2020)
Salvianolic Acid B	HeLa cells	Reduce the expression level of TGF	Time-effect, dose-effect	Jing et al. (2016)
Praeruptorin-B	HeLa and Siha cells	Down-regulated the expression of NF-κB, MMP-2 and MMP-9	Dose-effect	Hung et al. (2019)

## 5 Natural Products for Cervical Cancer in Clinical Trails

### 5.1 Paclitaxel

To date, Neoadjuvant chemotherapy has been tried to treat patients with advanced cervical cancer (Benedetti-Panici et al., 2002). *Paclitaxel,* a terpenoid compound, has become a commonly used chemotherapeutic agent and is usually used with cisplatin (Sofias et al., 2017). Regarding response rate (RR) and progression-free survival (PFS), numerous data indicate that cisplatin combined with *paclitaxel* has better efficacy and safety than cisplatin alone. Unsurprisingly, cisplatin combined with *paclitaxel* and bevacizumab has become the first-line treatment for metastatic cervical cancer (Marth et al., 2017). Some studies have also investigated the effect of paclitaxel in neoadjuvant therapy.

Tambaro et al. performed surgery on 42 patients with cervical cancer, 32 of whom were treated with *paclitaxel*, cisplatin, and epirubicin (CEP) as adjuvant therapy. The results found complete remission in 8 cases (25%), partial remission in 17 cases (53%), and stable disease in 9 cases (28%). Similarly, the RR was 78.5% at the end of chemotherapy (Tambaro et al., 2004).

Moreover, other chemotherapeutic agents in combination with *paclitaxel* are also being studied. For example, carboplatin combined with *paclitaxel* also had better tolerability and higher RR (Meletios et al., 2002). Pectaside et al. studied 51 patients with advanced cervical cancer, and when they were treated with paclitaxel and carboplatin, 16 percent had complete remission and 37 percent had partial remission. Further, the relative risk for patients who received only radiotherapy was 68%, while the relative risk for chemotherapy patients was only 28% (Pectasides et al., 2008). The British Columbia (BC) Cancer Institute in Vancouver stated that the combination of carboplatin and *paclitaxel* is the standard treatment for advanced cervical cancer, The combination of carboplatin and *paclitaxel* has a higher RR and better PFS than cisplatin alone (Meletios et al., 2002). Takekuma et al. also studied the combination of *paclitaxel* and nedaplatin in treating patients with advanced cervical cancer. Takekuma et al. evaluated the efficacy of *paclitaxel* combined with nedaplatin intreating 50 patients with cervical cancer. Discontinue co-therapy when disease progression changes or adverse reactions occur (Sutton et al., 1993; Monk et al., 2009; Takekuma et al., 2011). A phase II trial study also investigated the role of paclitaxel as monotherapy to treat patients with advanced cervical cancer. After treatment, the RR was 17 percent (Mcguire et al., 1996).

Collectively, the combination of paclitaxel with some chemotherapeutic agents such as platinum has a better effect (Della Corte et al., 2020). However, given the primary and acquired resistance to *paclitaxel*, molecules that increase the sensitivity of cancer cells to *paclitaxel*, like transmembrane-associated multidrug resistance proteins (P-GP, MRP-1 and ABCG2), could be selected. This also indicates that *paclitaxel* still has value in clinical application.

### 5.2 Curcumin

*Paclitaxel* has the function of inducing radiosensitization. When these natural products are combined with chemotherapeutic agents such as platinum drugs, the effect of radiotherapy can be better played - the ability to control the growth and metastasis of cancer cells is greatly enhanced. It is important to note that the enhancement of chemotherapy effect has a severe challenge to focus on: the amplification of cytotoxicity of chemotherapy. It is necessary to introduce a product that can induce chemotherapeutic agents without harming normal tissue as much as possible and increase sensitivity to chemotherapeutics. *Curcumin* is a classic example (Lao et al., 2006; Javvadi et al., 2008; Li et al., 2017).

According to a study by Javvadi et al., *curcumin* enhances the antioxidant effect of normal cells by increasing ROS production in cancer cells and down-regulates the NF-κB and AKT signaling pathways, thereby enhancing the re-sensitization of radiotherapy-resistant cells (Javvadi et al., 2008). Another finding was that thioredoxin reductase 1 (TxnRd1) could be induced by *curcumin*, and the sensitivity of cancer cells to radiation therapy is increased. TxnRd1, an antioxidant enzyme, may remove the concentration of intracellular ROS produced by infrared radiation. Overexpression of TxnRd1 could enhance the anti-sensitivity effect of cancer cells (Javvadi et al., 2010). *Curcumin* could increase the sensitization of cancer cells to radiation drugs and radiation.

In addition, a Phase I clinical study conducted by Cheng et al. (2001) in which 4 patients with cervical intraepithelial neoplasia were treated with a dose of curcumin, biopsies and analysis were performed after 3 months. The results showed that histological improvement in two patients. The main weakness of the research is that there were few patients involved, and one patient’s condition worsened. The clinical effects of *curcumin* are still of great value and potential. At present, *curcumin* tends to be used with other chemotherapeutic agents in order to achieve a better therapeutic effect.

## 6 Technologies to Improve the Anticancer Properties of Natural Products

Currently, radiotherapy and chemotherapy are commonly used for cancer treatment (Falzone et al., 2018), but they have specific negative effects. Targeted cancer therapy enables the precise treatment of molecules unique to particular cancer (Troy, 2015). However, this represents a high degree of dependence of targeted therapies on cancer-related mutant proteins and signaling pathways. Suppose the targeted gene mutation or signal pathway changes, the efficacy of targeted therapy will be greatly reduced. It shows that due to the abnormal physiological mechanism of cancer cells, the drug resistance of cancer cells to anti-cancer drugs is a problem that cannot be ignored. Cancer cells have the ability to adapt flexibly to various mechanisms to increase resistance to anti-cancer drugs, such as increased immune evasion, increased gene mutations, reactivation of drug targets, and altered metabolism (Ma and Zong, 2020). As a result, most anticancer drugs have a good effect in the initial treatment, but the efficacy is weakened after drug resistance or even relapse after chemotherapy. At present, the anticancer application of natural products is mainly reflected in the use of natural products to improve the resistance of cancer cells to cancer treatment, so it is necessary to consider improving the bioavailability of natural products (Liskova et al., 2021a).

### 6.1 Combined Use of a Variety of Natural Products

At present, there are few natural chemotherapeutic drugs directly used in the clinic, but the resistance of cancer cells to chemotherapeutic drugs is a problem that must be faced. The current study found that natural products can increase the efficacy of natural chemotherapeutic drugs by sensitizing cancer cells to them again.

*Paclitaxel* has become a commonly used chemotherapy drug, and some studies have proved that the combination of *curcumin* and *paclitaxel* can improve the efficacy of *paclitaxel*. Combined treatment with liposome curcumin and *paclitaxel* synergistically reduced the size and incidence of squamous cervical cancer models in mice compared with monotherapy (Sreekanth and Bava, 2011). Further studies have shown that curcumin regulates NF-κB pathway related proteins and genes and regulates the activities of c-Jun N-terminal kinase (JNK) and extracellular regulated protein kinases (ERK), thus synergistically improving the efficacy of combination therapy with paclitaxel. In addition, another report showed that the combination of paclitaxel and curcumin had a more potent anticancer response than paclitaxel alone, targeting HeLa cells (Bava et al., 2004). Similar to curcumin, the combination of quercetin and docetaxel effectively slowed tumor growth. Quercetin can also induce ROS production, thereby promoting the effect of paclitaxel on prostate cancer PC-3 cells (Zhang et al., 2020). These undoubtedly show that the combination of natural products can increase the sensitization of cancer cells.

### 6.2 Using Nanotechnology to Promote the Anticancer Effects of Natural Products

Although natural products have sound anti-cancer and sensitizing effects, the premise of maximizing the effects of natural products is to improve the bioavailability of natural products. At present, nanotechnology can be used to optimize the rigid limitations such as the bioavailability of natural products to improve their therapeutic efficacy and pharmacokinetic characteristics (Masashi et al., 2011; Prasad et al., 2014; Sahebkar and Momtazi, 2016; Ahmed et al., 2017a). Nanotechnology can be used to modify and encapsulate natural products, thus giving natural products a longer half-life and enhancing their targeting ability. Different types of nanotechnology have been widely used to compensate for the shortcomings of natural products.

#### 6.2.1. Polymeric Nanoparticles

PNPs are nanospheres and nanocapsules that exist in a solid phase and have different structures and compositions (Ahmed et al., 2021). The nanocapsules have an oily core encased in a polymer film on which natural products can be adsorbed and dissolved. Nanospheres are a matrix system on which natural products can be directly retained (Alexis et al., 2008). PNPs could control drug release and targeted therapy and act on the cell surface to enhance the therapeutic index (Fonseca-Santos and Chorilli, 2020). Typical polymer nanoparticles include chitosan, poly (lactic-coglycolic acid) (PLGA) nanospheres, and poly (butyl cyanoacrylate) (PBCA).

Chitosan is a linear polysaccharide with good biocompatibility, low toxicity and biodegradability. At present, chitosan has been widely used in nanomedicine and has great potential in cancer treatment (Dev et al., 2010; Jin et al., 2016; Kaur et al., 2020). Studies have found that the combination of gambogic acid and chlorthalidone retinoic acid supported ethylene glycol chitosan nanoparticles can effectively inhibit the growth of osteosarcoma MG63 cancer cells (Liu et al., 2016). In addition, in another study of metastatic melanoma in the lungs, chitosan nanoparticles loaded with curcumin inhibited tumor nodules three times more than free curcumin did (Havel et al., 2016). PLGA is a biodegradable functional polymer organic compound synthesized by the polymerization of lactic acid and glycolic acid. PLGA nanoparticles are widely used in pharmaceutical, medical engineering materials and modern industry due to their excellent biocompatibility, non-toxicity and exemplary performance in forming capsules and films (Ahmed et al., 2021). PLGA could also enhance the anticancer effect of natural products. Mukherjee et al. found that curcumin-PLGA nanospheres produced a more significant anticancer effect on prostate cancer cells (Mukerjee and Vishwanatha, 2009). Yallapu et al. also demonstrated that curcumin-PLGA nanoparticles were more effectively internalized into prostate cancer cells than free curcumin (Yallapu et al., 2014; A.; Aljuffali et al., 2016). PBCA nanoparticles are also ideal for transporting natural products (Rempe et al., 2014). PBCA nanoparticles can effectively prevent the degradation of natural products. One study found that the half-life of PBCA nanoparticles loaded with curcumin was 52 times increased by intravenous administration, while the final scavenging amount of curcumin was reduced by 2.5 times (Duan et al., 2010). In addition, PEGylated nanoparticles coated with curcumin have a slower release rate. Compared with free curcumin, the size of the tumor treated with PEGylated nanoparticles coated with curcumin was 2.7 times smaller (A. Aljuffali et al., 2016), and PEGylated nanoparticles did not cause significant toxicity.

#### 6.2.2. Lipid Nanoparticles

PNPs are a typical drug transport carrier, but polymer toxicity and solvent residues after synthesis are the problems we have to face when using PNPs (Blomberg et al., 2001). LNPs can effectively avoid these problems (Muller and Keck, 2004). Solid lipid nanoparticles (SLNs) are nanospheres composed of Solid lipid cores that can control drug release, avoid drug degradation or leakage, and have good targeting properties. At present, SLNs can be mass-produced by the high-pressure milk homogenization method. Other preparation methods are the emulsification precipitation method and microemulsion method (Kumar et al., 2012; Kashyap et al., 2021). It has been confirmed that SLNs can improve the anticancer activity of natural products. Vandita et al.'s study concluded that SLNs coated with curcumin significantly reduced IC_50_ against human cancer cells compared to capcurcumin (Vandita et al., 2012).

#### 6.2.3. Protein Nanoparticles

Protein is one of the best materials for the synthesis of nanoparticles (Figueiró et al., 2013). The surface of protein nanoparticles can be well modified and connected to natural products (Mukherjee et al., 2011). At the same time, protein nanoparticles are biodegradable, which makes protein nanoparticles be considered for various drug therapy.

Gelatin nanoparticles are widely used in drug delivery due to their high biocompatibility. It has been found that gelatin nanoparticles loaded with resveratrol are more likely to be taken up by cells (Signorelli and Ghidoni, 2005). Similarly, paclitaxel-gelatin nanoparticles have good anti-bladder cancer activity (Lu et al., 2004). As protein nanoparticles, albumin nanoparticles can be modified with resveratrol. In a study for ovarian cancer, using resveratrol -albumin nanoparticles significantly increased the drug’s accumulation in the ovaries and reduced the drug’s concentration in the blood compared with free resveratrol injections. It also highlights the excellent role of albumin nanoparticles in drug encapsulation and targeted transport (Guo et al., 2010). In addition, an albumin nanoparticle formulation containing paclitaxel has been approved for the treatment of patients with metastatic breast cancer (Desai et al., 2006).

#### 6.2.4. Metal Nanoparticles

In the past, the synthesis of metal and metal oxide nanoparticles has been extensively studied by researchers, and there have been continuous attempts to apply metal nanoparticles to biomedicine, and particular achievements have been achieved (Ahmed et al., 2016b). However, the process of synthesizing metal nanoparticles through physical and chemical synthesis is cumbersome and has the potential to pose risks to the environment, as the synthesis requires a variety of hazardous chemicals and hazardous substances. Direct use of these metal nanoparticles on humans may also have negative effects such as toxicity. Biosynthesis of metal nanoparticles is a simple, safe and green way of synthesis. In biosynthesis, various parts of plants and fungi can synthesize metal nanoparticles (Ahmed et al., 2016a; Ahmed et al., 2016b) and adjust the size and shape of nanoparticles because biologically active compounds in plants can accelerate the conversion of metal ions into biologically active nanoparticles (Munir et al., 2021). The researchers took note of biosynthesis and investigated the effects of plant-based metal nanoparticles.

Plant-synthesized gold nanoparticles have unique physicochemical properties and biocompatibility, and are widely used in antibacterial therapy, drug delivery and photothermal therapy (Begum et al., 2022; Habeeb Rahuman et al., 2022). (Shukla et al., 2012) treated prostate cancer with EGCG as a carrier of gold nanoparticles and found that gold nanoparticles loaded with EGCG reduced tumor size by 80% without toxicity after injection of the drug in xenograft mice (R. et al., 2012). In addition, compared with free EGCG, the IC_50_ value of nano-EGCG for bladder cancer cell lines was 6–7 times higher than that of free EGCG (Hsieh et al., 2011). Silver nanoparticles are a good antibacterial agent, and it has been found that modified plant silver nanoparticles can be used in cancer treatment (Habeeb Rahuman et al., 2022). The leaf of *Taraxacum abietifolium* Saarsoo extract used in conjunction with silver nanoparticles showed good anticancer activity against liver cancer cells, and it is worth noting that this anticancer activity is comparable to commercial anticancer drugs (Gauthami et al., 2015). Similar to most metal nanoparticles, zinc oxide nanoparticles have good antibacterial effects (Begum et al., 2022). Biosynthesized ZnO nanoparticles have more advantages than physical and chemical synthesized ZnO nanoparticles (Ahmed et al., 2017b). Doxorubicin combined with ZnO nanoparticles synthesized from fruit extracts is more cytotoxic than doxorubicin alone in the treatment of breast and colon cancer cells (Guo et al., 2010).

#### 6.2.5. Other Nanotechnologies

In addition to nanoparticles, other nanotechnologies such as liposomes and dendrimers are also frequently used to diagnose and treat cancer. Liposomes are tiny spherical vesicles with bilayer phospholipids and cholesterol and are amphiphilic (Temidayo et al., 2018). Liposomes provide excellent protection against drugs and reduce the nonspecific toxicity associated with natural products. It is noteworthy that liposomes are capable of delivering packaged drugs directly into cells, and these characteristics make liposomes frequently used for targeted drug delivery (Barazza et al., 2005; Parhi et al., 2012; Hwang et al., 2022). Dendrimers are composed of degradable monomers. Dendrimers are not toxic by themselves, and their internal cavities and binding sites are capable of carrying drugs. In addition, there are a lot of functional groups on the surface of Dendrimers, and the modified dendrimers have a good targeting effect (Kaur et al., 2020). Many research teams have begun to use dendrimers to wrap natural products and study the anticancer effects of the complex. For example, Babaei et al. have tried to wrap curcumin into dendrimers and use the complex to treat fibrosarcoma (Babaei et al., 2012).

## 7 Conclusion and Future Perspectives

HPV is a necessary factor contributing to the occurrence of cervical cancer. So blocking the oncogene expression of HPV from the beginning is a treatment idea. Many abnormal mechanisms, including further amplification of inflammation, are driven by oncoprotein expression. This review illustrates the view that inflammation plays an important role inregulating both the onset and end of cancer. Immunotherapy has become a hot topic in cancer treatment today because of a broad understanding of the relationship between inflammation and cancer. Further study of these mechanisms may contribute to the application of immunotherapy. However, this process is lengthy. In addition to selecting typical biomarkers, clinical trials and retrospective studies of these biomarkers are also required. It should not be ignored that attention should also be paid to matching the selected models with human tumors when selecting preclinical models.

Plants and animals, marine life and even microbes are a treasure trove of natural products notable for their diversity and low toxicity. At present, natural products are mainly used as sensitizers in cancer treatment. However, the anticancer potential of natural products should not be ignored, considering their limitations and whether there is a possibility to solve the drug resistance of cancer cells. In addition, it is worth thinking that the microbiota is closely related to inflammation. Intestinal microbes can also indirectly affect the microbiota and hormone changes in the reproductive tract, thereby regulating inflammation and affecting the occurrence of cervical cancer. So the link between natural products and the gut microbiome may be an emerging field. Fortunately, it has been found that berberine can directly interact with intestinal microorganisms to improve metabolic disorders and inhibit oxidation and inflammatory mediators. Further studies combining pharmacodynamics, pharmacokinetics, microbiome and metabonomics are recommended to develop new therapies for the prevention and treatment of cervical cancer.

However, there are still disadvantages to developing and using natural products. The characteristics of low absorption, extensive metabolism and metabolic elimination need to be addressed. Besides, some natural products require sufficiently high concentrations to reach the target tissue effectively. Toxicity is also a fact that cannot be ignored. In the face of these limitations, screening and optimization methods are needed. At present, the value of natural products can be developed by optimizing the solubility and bioavailability of natural products. Firstly, the lead compound is modified and modified by means of medicinal chemistry. Secondly, choose a suitable carrier to optimize the delivery of natural products. Nanoparticles are a good example, and more attempts have been made. Nanoparticles are carriers with great potential. It is worth considering that although nanoparticles have the ability to improve drug bioavailability significantly, there are too few cases that have entered clinical trials. Different delivery systems have different advantages and disadvantages. In addition to choosing a delivery carrier that better matches the designated natural product, biocompatibility and safety should also be considered. Researchers may need to spend more effort exploring the ways and mechanisms of drug delivery. We believe that in addition to improving the delivery mode of the carrier itself, we may also start from external media to improve the conditions of the surrounding environment to improve the accuracy of the delivery of natural products. In addition, combination with other drugs is a way to increase the sensitivity of cancer cells. However, due to the multi-effect characteristics of natural products, drug combinations may have interaction effects, so the possible risks and benefits derived from the combined use of natural products need to be grasped. Another interesting approach is taking food and ysing natural products as dietary supplements to achieve therapeutic effects. Phenolic compounds, for example, are found in vegetables, fruits and nuts. This method could also effectively avoid the toxic effects caused by high concentrations. However, the effect of this method is limited, and the daily nutrition for the body can be used in this way, while the treatment of cancer still has to rely on radiotherapy and chemotherapy. Bioavailability needs to be rethought. The effectiveness and safety of long-term use also need to be demonstrated. It is worth noting that the efficiency of absorption, distribution, kidney excretion and liver elimination of the active ingredients in natural products is usually unknown in the human body, and only the conclusions and experience of *in vitro* and animal experiments are insufficient to infer the detailed effects of natural products *in vivo* accurately.

Collectively, detailed information on the pharmacology, formulation, potential toxicity and side effects of a drug needs to be available before it can be used. Although this review aims to demonstrate that natural products are a promising anti-cervical cancer drug, cancer is an extremely complex disease and treatment with natural products alone may not be sufficient to cure cancer completely. It is recommended that combination therapies and delivery systems be used simultaneously, with detailed information, to overcome the challenges of drug resistance and side effects. In addition to pre-clinical studies, clinical studies are also necessary for continuous validation. It takes a combined effort of cancer researchers, chemists and clinical researchers.

As discussed above, many natural products suffer from two major drawbacks: low bioavailability and toxicity, both of which require researchers to find further solutions. Natural products not only have effects that are relatively easy to obtain and low toxicity, but more importantly as a gold mine, which could bring great exploration value because of diversity and complexity. It is still of excellent significance to study the role of natural products in treating cancer.

## Finding

This work was supported by the Changsha Central Hospital Affiliated to University of South China Foundation of key Program (YNKY201901), Hunan Province Foundation of High-level Health Talent (225 Program), and Science and Technology Key Program of Hunan Provincial Health Committee (20201904), Natural Science Foundation of Hunan Province (2021JJ30753).

## Glossary

ADP: adenosine diphosphate
CSCs: cancer stem cells
CAT: catalase
CDA: cytidyl deaminase
COX-2: cyclooxygenase-2
E6AP: E6-associated protein: E6-associated protein
EMT: epithelial-mesenchymal transition
E6AP: E6-associated protein: E6-associated protein
EGCG: Epigallocatechin gallate
ERK: extracellular regulated protein kinases
FAK: focal adhesion kinases
GSH: glutathione
HPV: human papilloma virus
HIF-1α: hypoxia inducible factor-1α
HSV: herpes simplex virus
IL: Interleukin
JNK: c-Jun N-terminal kinase
LNPs: Lipid nanoparticles
MMP: matrix metalloproteinase
NO: nitric oxide: nitric oxide
NF-κB: nuclearfactor-kappaB
NO: nitric oxide: nitric oxide
PNPs: Polymeric Nanoparticles
PLGA: poly (lactic-coglycolic acid)
PBCA: poly (butyl cyanoacrylate)
ROS: reactive oxygen species
Rb: retinoblastoma protein
SOD: superoxide dismutase
STAT3: signal transducer and activator of transcription
SLNs: Solid lipid nanoparticles.
TAMs: tumor-associated macrophages
TNF-α: tumor necrosis factor-α
TGF: transforming growth factor
TME: tumor microenvironment
TxnRd1: thioredoxin reductase 1
